# Localization of TRPV3/4 and PIEZO1/2 sensory receptors in murine and human larynges

**DOI:** 10.1002/lio2.968

**Published:** 2022-11-02

**Authors:** Alexander G. Foote, Julianna Tibbetts, Stephanie M. Bartley, Susan L. Thibeault

**Affiliations:** ^1^ Division of Otolaryngology – Head and Neck Surgery University of Wisconsin – Madison Madison Wisconsin USA

**Keywords:** development, larynx, sensory receptors, somatosensation, vocal fold

## Abstract

**Objective:**

The primary aim of this study was to identify expression of TRPV3 and TRPV4 chemoreceptors across perinatal and adult stages using a murine model with direct comparisons to human laryngeal mucosa. Our secondary aim was to establish novel cell expression patterns of mechanoreceptors PIEZO1 and PIEZO2 in human tissue samples.

**Study design:**

In vivo*.*

**Methods:**

We harvested murine laryngeal tissue to localize and describe TRPV3/4 endogenous protein expression patterns via immunofluorescence analyses across two developmental (E16.5, P0) and adult (6 weeks) timepoints. Additionally, we obtained a 60‐year‐old female larynx including the proximal trachea and esophagus to investigate TRPV3/4 and PIEZO1/2 protein expression patterns via immunofluorescence analyses for comparison to murine adult tissue.

**Results:**

Murine TRPV3/4 expression was noted at E16.5 with epithelial cell colocalization to supraglottic regions of the arytenoids, aryepiglottic folds and epiglottis through to birth (P0), extending to the adult timepoint. Human TRPV3/4 protein expression was most evident to epithelium of the arytenoid region, with additional expression of TRPV3 and TRPV4 to proximal esophageal and tracheal epithelium, respectively. Human PIEZO1 expression was selective to differentiated, stratified squamous epithelia of the true vocal fold and esophagus, while PIEZO2 expression exhibited selectivity for intermediate and respiratory epithelia of the false vocal fold, ventricles, subglottis, arytenoid, and trachea.

**Conclusion:**

Results exhibited expression of TRPV3/4 chemoreceptors in utero, suggesting their importance during fetal/neonatal stages. TRPV3/4 and PIEZO1/2 were noted to adult murine and human laryngeal epithelium. Data indicates conservation of chemosensory receptors across species given similar regional expression in both the murine and human larynx.

## Graphical abstract

Sensory receptors of the larynx.

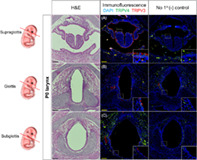

## 1 INTRODUCTION

The larynx is a complex organ that has an array of functions that are important for normal activation of reflexes associated with breathing, airway protection, and phonation across the lifespan.^1^, ^2^, ^3^, ^4^, ^5^, ^6^ Several reflexes, collectively known as the laryngeal chemoreflex (LCR), are initiated in the fetus and newborn when hypochloremic or acidic solutions contact the epithelium—thought to be feto‐protective during perinatal development.^7^, ^8^ LCR requires continuous monitoring of the mucosal environment throughout life via cellular and neural laryngeal sensory receptors. However, expression of sensory receptors during developmental stages remains unknown. Structural and functional changes to cells and tissues during perinatal development provide a critical period for maturation of chemoreceptors with important implications for pediatric dysphagia^9^ and laryngeal neurodevelopmental abnormalities.^10^ Defining temporal and spatial specificity of sensory receptors across development may help better understand the mechanism(s) behind the LCR and its reflex arc across the lifespan.

Ion channels of the transient receptor potential (TRP) superfamily are involved in a wide variety of neural signaling processes, most notably in sensory receptor cells, and have been implicated in chemosensation,^11^, ^12^, ^13^, ^14^, ^15^ in addition to their mediated role in mechanotransduction.^16^, ^17^, ^18^, ^19^, ^20^, ^21^, ^22^ While much is to be uncovered, recent progress into laryngeal somatosensation has localized TRP vanilloid (V) channels 1–4 within adult laryngeal epithelium of mice and humans,^13^, ^15^ P2X3 type adenosine triphosphate receptors in type III taste bud cells within murine arytenoid tissue,^23^ vagal P2RY1 neural receptors innervating the murine larynx,^24^ and PIEZO1 mechanoreceptor in murine laryngeal epithelia.^25^ TRPV channels have also been hypothesized to act as laryngeal nociceptors associated with pathological conditions, such as inflammatory response, genesis of cough and asthma.^14^, ^15^ While their assumed importance for chemosensation in adult laryngeal mucosa has been established,^13^, ^15^ investigation into developmental expression of these essential chemoreceptors is warranted given the importance of LCR in the fetus and newborn.

The primary aim of this study was to identify expression of TRPV3/4 at E16.5, P0 and adult 6 weeks stages using a murine model. We chose to investigate TRPV3/4 channels given their integrated importance in another sensitive cell type (e.g., skin keratinocytes),^26^ and *Trpv4* reported association with *Piezo1* mechanoreceptor.^18^, ^19^, ^21^, ^27^ Additionally, we localized TRPV3/4 in the murine adult larynx for direct comparison to adult human mucosal tissue. TRPV3, activated by temperature (>31°C), has been found expressed in dorsal root ganglion (DRG), skin, testis, stomach, small intestine, trachea, and placenta with suspected physiological roles for thermal and pain sensation.^13^, ^15^ TRPV4, activated by temperature (>27°C), has been found expressed in DRG, kidney, skin, inner ear, heart, brain, endothelium, hypothalamus, trachea, and lung with physiological roles for CNS osmotic regulation, thermal preference and pain sensation.^13^, ^15^, ^28^, ^29^ Our secondary aim was to establish novel cell expression of PIEZO1/2 in human tissue. While behavioral and electrophysiological human studies have implicated mechanosensory receptors in laryngeal mucosa,^4^, ^5^ localization of *Piezo* mechanoreceptors is unknown. *Piezo* mechanoreceptors function in cells to convert mechanical forces into biological signals.^30^ *Piezo1* has been previously localized to epithelia^31^ and shown to regulate the life cycle of these cells.^32^, ^33^ *Piezo2* has been found most often associated with neurons and has important roles for sensory perception^34^ and integration of mechanical and thermal cues.^35^ We chose to investigate mucosal tissue types known to trigger airway protective response reflexes (i.e., arytenoid cartilage, proximal trachea), exhibit little to no reflex response (i.e., true vocal fold), and epithelium of similar morphology and developmental origins (i.e., proximal esophagus).

Identity, functions and response properties of various sensory targets at the laryngeal mucosal‐airway interface have been limited, with ambiguity regarding species comparisons for translational impact. In this study, we localized TRPV3/4 chemoreceptors to murine epithelium across developmental stages in addition to characterizing novel PIEZO1/2 mechanoreceptors in human laryngeal mucosa. Insight into laryngeal sensory receptors may help to better explain LCR abnormalities observed in the pathogenesis of certain fetal/neonatal (prolonged apnea, sudden infant death syndrome)^8^, ^36^ and adult sensory‐based disorders (chronic cough, dysphagia, laryngospasm).^37^, ^38^, ^39^, ^40^

## 2 MATERIALS AND METHODS

### 2.1 Mouse and human tissue collection

**FIGURE 1:**
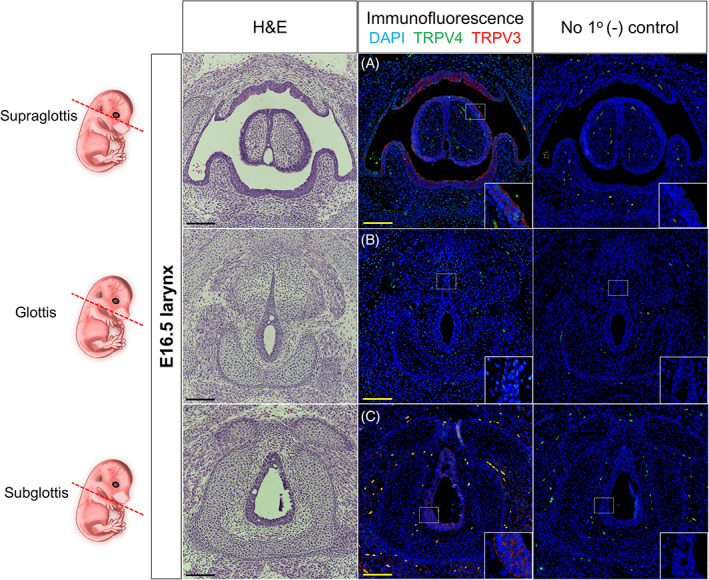
Laryngeal TRPV3/4 chemoreceptors at embryonic (E) day 16.5. Schematic of embryonic laryngeal sections (red dotted line) for respective images. Hematoxylin and eosin‐stained and immunofluorescent‐stained murine samples for TRPV3 (red) and TRPV4 (green) denoting supraglottic, glottic, and subglottic transverse serial sections. (A) TRPV3/4 colocalized to epithelia of the superior arytenoid, posterior glottis and inferior aspect of laryngeal side of the epiglottis. (B) TRPV4 expression exhibited to vocal fold epithelia and surrounding mesenchymal cells, albeit, with no TRPV3 expression. (C) TRPV3 expression exhibited to subglottic epithelial cells, albeit, no TRPV4 expression. (A–C) Negative controls lack antiserum expressivity. DAPI is in blue. All images taken at 30× magnification with insets denoted by white dotted boxes. Scale bar represents 100 μm for all images. E, embryonic

**FIGURE 2:**
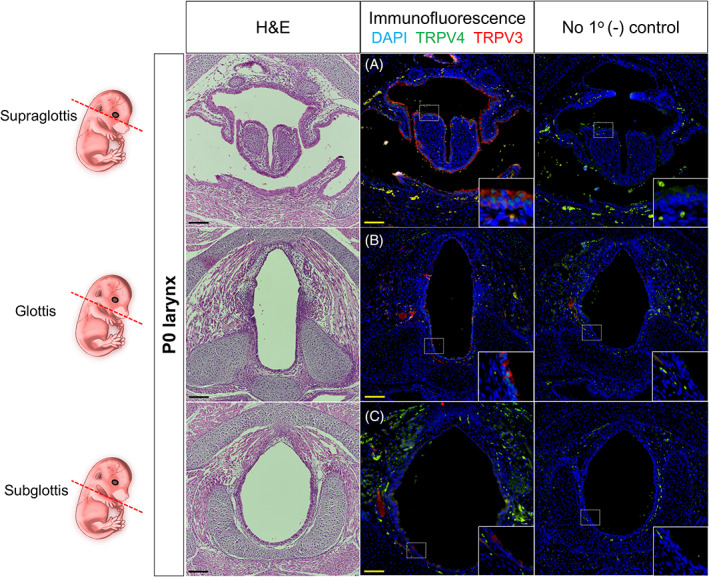
Laryngeal TRPV3/4 chemoreceptors at postnatal (P) day 0. Schematic of embryonic laryngeal sections (red dotted line) for respective images. Hematoxylin and eosin‐stained and immunofluorescent‐stained murine samples for TRPV3 (red) and TRPV4 (green) denoting supraglottic, glottic, and subglottic transverse serial sections. (A) TRPV3/4 colocalized to epithelia of the superior arytenoid, and anterior/posterior supraglottis. (B) TRPV3/4 colocalized expression to epithelia of the posterior glottis, with minimal expression to vocal fold epithelia. (C) TRPV3 expression exhibited to sparce epithelial cells of the posterior subglottis, albeit, no TRPV4 expression. (A–C) Negative controls lack antiserum expressivity. DAPI is in blue. All images taken at 20× magnification with insets denoted by white dotted boxes. Scale bar represents 100 μm for all images. E, embryonic

**FIGURE 3:**
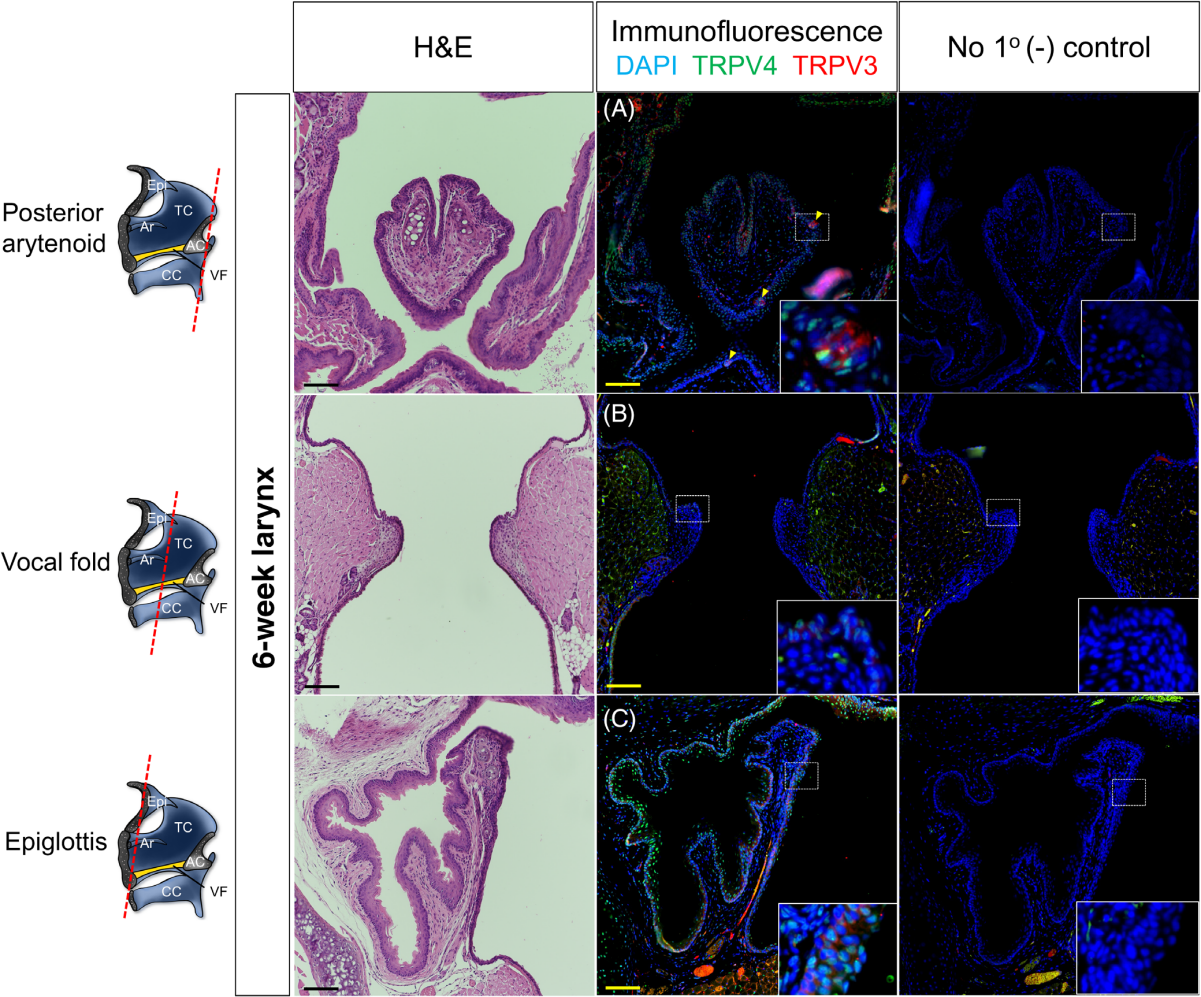
Laryngeal TRPV3/4 chemoreceptors at the adult 6‐week timepoint. Schematic of laryngeal sections (red dotted line) for respective images. Hematoxylin and eosin‐stained and immunofluorescent‐stained murine samples for TRPV3 (red) and TRPV4 (green) denoting posterior arytenoid region, vocal fold, and epiglottic coronal serial sections. (A) Extensive TRPV4 expression to epithelia of the posterior glottis, with colocalized TRPV3/4 expression restricted to taste buds (yellow arrows). TRPV3 exhibits basal cell preferential expression patterns. (B) TRPV3/4 colocalized expression to epithelia of the subglottis, with minimal expression to vocal fold epithelia. (C) Extensive colocalized TRPV3/4 expression to the epiglottis and hypopharynx, with TRPV3 exhibiting basal cell preferential expression. (A–C) Negative controls lack antiserum expressivity. DAPI is in blue. All images taken at 20× magnification with insets denoted by white dotted boxes. Scale bar represents 100 μm for all images. AC, arytenoid cartilage; Ar, aryepiglottic fold; CC, cricoid cartilage; Epi, epiglottis; TC, thyroid cartilage; VF, vocal fold

C57BL/6J (#000664) mice were obtained from The Jackson Laboratory (Bar Harbor, Maine). Laryngeal tissue was collected at the following time points: embryonic day (E) 16.5, postnatal day (P) 0, and adult (6 weeks) and immediately fixed in 4% paraformaldehyde in phosphate‐buffered saline (PBS) at 4°C overnight. At least three embryos, pups or adult animals were examined at timepoints shown in Figures 1, 2, 3. Pregnant dams and adults were sacrificed for experiments through CO_2_ asphyxiation and cervical dislocation followed by isolation of vital organs (larynx) and embryos. Fetal and neonate mice (<10 days) were put on ice until motionless and euthanized via decapitation. Timed pregnancies were confirmed through visualization of vaginal plugs with noon on the day vaginal plugs were detected designated as E0.5. Human larynx of post‐mortem 60‐year‐old female was obtained from the National Disease Research Interchange (Philadelphia, PA). Human tissue was procured from donor within 18 h of death and was received within 48 h of death fixed in 10% formalin. Sample was subsequently fixed in 10% formalin for up to 1 month at room temperature (RT). Criteria for inclusion included subjects between 18 and 65 years of age, and larynges that were unaffected from any disease processes identified from medical report and visual inspection. Criteria for exclusion consisted of medical report of sepsis. Murine larynges from each timepoint were excised and treated appropriately for preservation following regulations of protocols approved by the University of Wisconsin Animal Care and Use Committee. Human larynx that included the proximal trachea and esophagus was obtained in accordance and with approval from the Institutional Review Board at the University of Wisconsin‐Madison. Mouse and human laryngeal tissue analyzed included the supraglottic (epiglottis, aryepiglottic folds, arytenoids, ventricular folds, and ventricles), glottic (vocal fold) and subglottic (trachea) regions.

### 2.2 Histology and immunofluorescence

Samples were subsequently dehydrated in a gradient series of ethanol, treated with xylene and embedded in paraffin wax. Paraffin wax blocks were cut into serial sections (5 μm), dewaxed and rehydrated, heated to boiling in 10 mM citrate buffer (pH 6) for antigen retrieval and treated with 0.5% Triton X‐100 in PBS for cell membrane permeabilization. Sections were then stained using a standard immunofluorescence (IF) protocol.^41^ IF staining for PIEZO2 proteins were performed using a direct Tyramide SuperBoost™ Signal Amplification (TSA) kit (B40943, Invitrogen) followed by TrueBlack® Lipofuscin Autoflourescence Quencher (Biotium) staining following product's published protocols. Negative controls included antiserum + block peptide, in addition to, no applied primary antiserum. Positive controls included DRG for TRPV3/4 and PIEZO2, and brain tissue for PIEZO1 (data not shown). All primary and secondary antibodies used are listed in Table 1. Primary antibodies were applied overnight at 4°C. Peptide block serum was mixed with either TRPV4 and/or PIEZO2 antibodies at 10‐to‐1 concentration ratio, preincubated for 60 min with rotation and then solution was applied overnight at 4°C. Sections were incubated with 4′,6‐diamidino‐2‐phenylindole (DAPI) (1:3000 ratio) for 10 min at RT. Slides were mounted and coverslipped with Prolong Diamond mounting media (Fisher P36970), cured flat at RT in the dark for 24 h, and stored at 4°C. Each experiment was replicated at least twice for all timepoints assessed.

**TABLE 1:** Antibodies used in this study

	Dilution	Supplier	Catalog #	Lot #
Primary antibodies				
Rabbit anti‐PIEZO1	1:100	Novus Biologicals	78446	B, C
Rabbit anti‐PIEZO2	1:200 (TSA)	Novus Biologicals	78624	E, F
Mouse, anti‐TRPV3	1:300	Abcam	ab85022	GR3249969‐21
Rabbit, anti‐TRPV4	1:400	Alomone Labs	ACC‐034	ACC034AN3050
Secondary antibodies				
Alexa Flour 488, Goat anti‐Rabbit IgG	1:500	ThermoFisher	A27034	UD2749322
Goat anti‐mouse IgG, Cy3™	1:200	Jackson Immuno Research	103‐165‐155	150532
Commercial Kits				
Alexa Flour 488, Tyramide SuperBoost	n/a	ThermoFisher	B40943	2291406

### 2.3 Image acquisition

Images were acquired on a Nikon Eclipse Ti2 inverted microscope with Nikon DS‐Ri2 camera and NIS Elements software. Images were uniformly adjusted for brightness and exposure using Nikon or ImageJ software. Larynx images were photographed at either 20× or 30× magnification. Sections from experimental groups were imaged using the same exposure settings or laser power for any given antibody combination. All schematic images were created in Procreate (version 5.2.2).

## 3 RESULTS

For murine developmental timepoints, three areas chosen for investigation included the supraglottis, glottis and subglottis. At stage E16.5, supraglottic expression of TRPV3/4 colocalized to epithelia of the arytenoid region, posterior glottis, and inferior aspect of laryngeal side of the epiglottis (Figure 1A). At the glottic level, TRPV4 was mildly expressed to vocal fold epithelia and surrounding mesenchymal cells at the anterior commissure, with no expression of TRPV3 (Figure 1B). At the level of the subglottis, TRPV3 localized to airway epithelial cells, with no expression of TRPV4 (Figure 1C). At stage P0, TRPV3/4 colocalized to the supraglottic epithelium, with additional colocalized expression to posterior esophageal epithelium (Figure 2A). At the level of the glottis, TRPV3/4 were largely restricted to epithelia of posterior regions, albeit, with mild expression to anterior epithelium (Figure 2B). At the level of the subglottis, TRPV3 was minimally localized in sparse epithelial cells within posterior regions, with no expression of TRPV4 (Figure 2C).

For the adult 6 weeks stage, we chose to investigate laryngeal mucosal regions known for increased sensory perception such as posterior arytenoid and epiglottis.^4^, ^5^ We also chose the mid‐membranous vocal fold region given its importance in tissue oscillation.^42^ At the posterior aspect of the arytenoid region, TRPV3/4 colocalized to taste buds, with robust TRPV4 epithelial cell expression (Figure 3A). TRPV4 was also noted to cells within submucosal glands. At the region of the vocal folds, weak expression of TRPV3/4 localized to vocal fold epithelia, however, strong expression was exhibited to the subglottic epithelium (Figure 3B). Lastly, at the anterior region of the epiglottis, TRPV3/4 exhibited robust expression to the epiglottic epithelium, with TRPV4 expression to epithelia of the hypopharynx and sparce positivity to surrounding mesenchymal cells. (Figure 3C).

**FIGURE 4:**
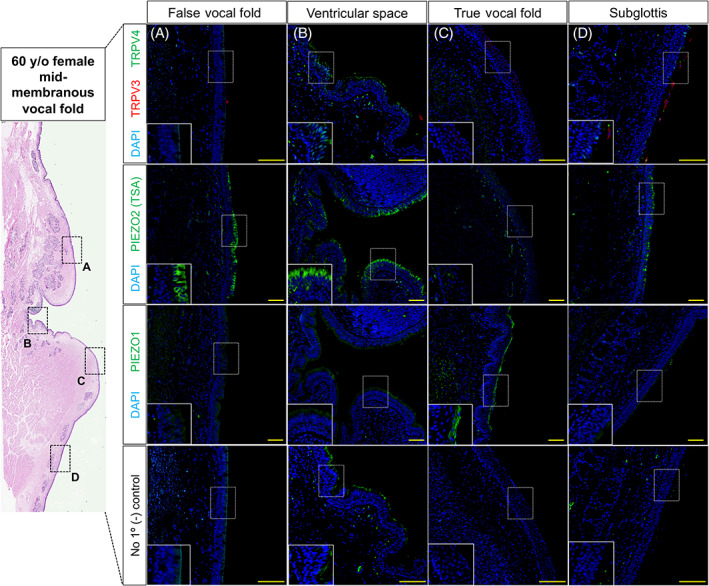
Mechano‐ and chemoreceptors in 60‐year‐old female mid‐membranous vocal fold tissue. Low magnification of hematoxylin and eosin‐stained coronal section. Immunofluorescence analysis for chemoreceptors TRVP3 (red) and TRPV4 (green) exhibited to epithelial cells of the ventricular space (B) and subglottic (D) regions. Mechanoreceptors PIEZO2 (green) exhibited to epithelial cells of the false vocal fold (A), ventricular space (B) and subglottic (D) regions, with PIEZO1 (green) expression to true vocal fold (C) apical epithelia only. Negative controls lack antiserum expressivity for all anatomic regions. DAPI is in blue. Images for TRPV3/4 taken at 30× and PIEZO1/2 at 20× magnification. Scale bar represents 100 μm for all images

**FIGURE 5:**
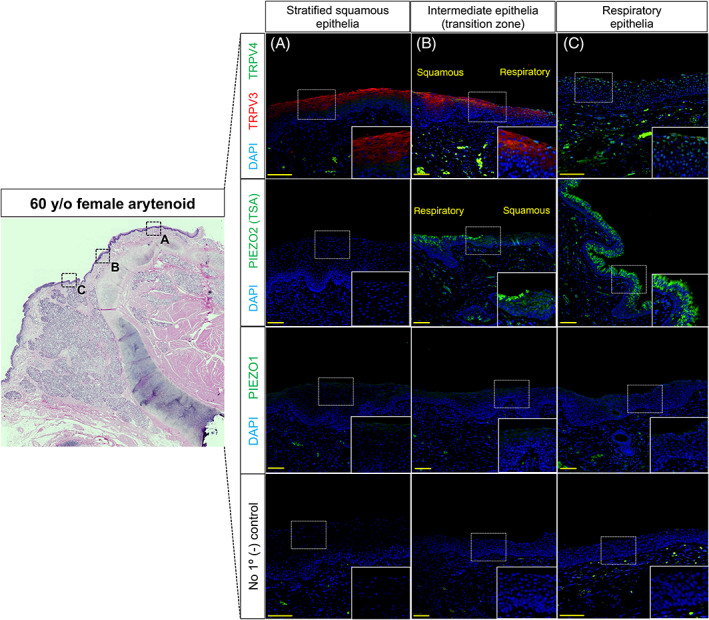
Mechano‐ and chemoreceptors in 60‐year‐old female posterior arytenoid tissue. Low magnification of hematoxylin and eosin‐stained coronal section of arytenoid region at the membranous‐cartilaginous junction. Immunofluorescence analysis for chemoreceptors TRVP3 (red) exhibited to stratified squamous (A) and intermediate epithelia (B) while TRPV4 (green) localized to respiratory epithelia only (B, C). Mechanoreceptors PIEZO2 (green) exhibited to respiratory epithelia only (B, C), with no PIEZO1 (green) expression to human arytenoid epithelium. Negative controls lack antiserum expressivity for all anatomic regions. DAPI is in blue. Images for TRPV3/4 taken at 30× and PIEZO1/2 at 20× magnification. Scale bar represents 100 μm

**FIGURE 6:**
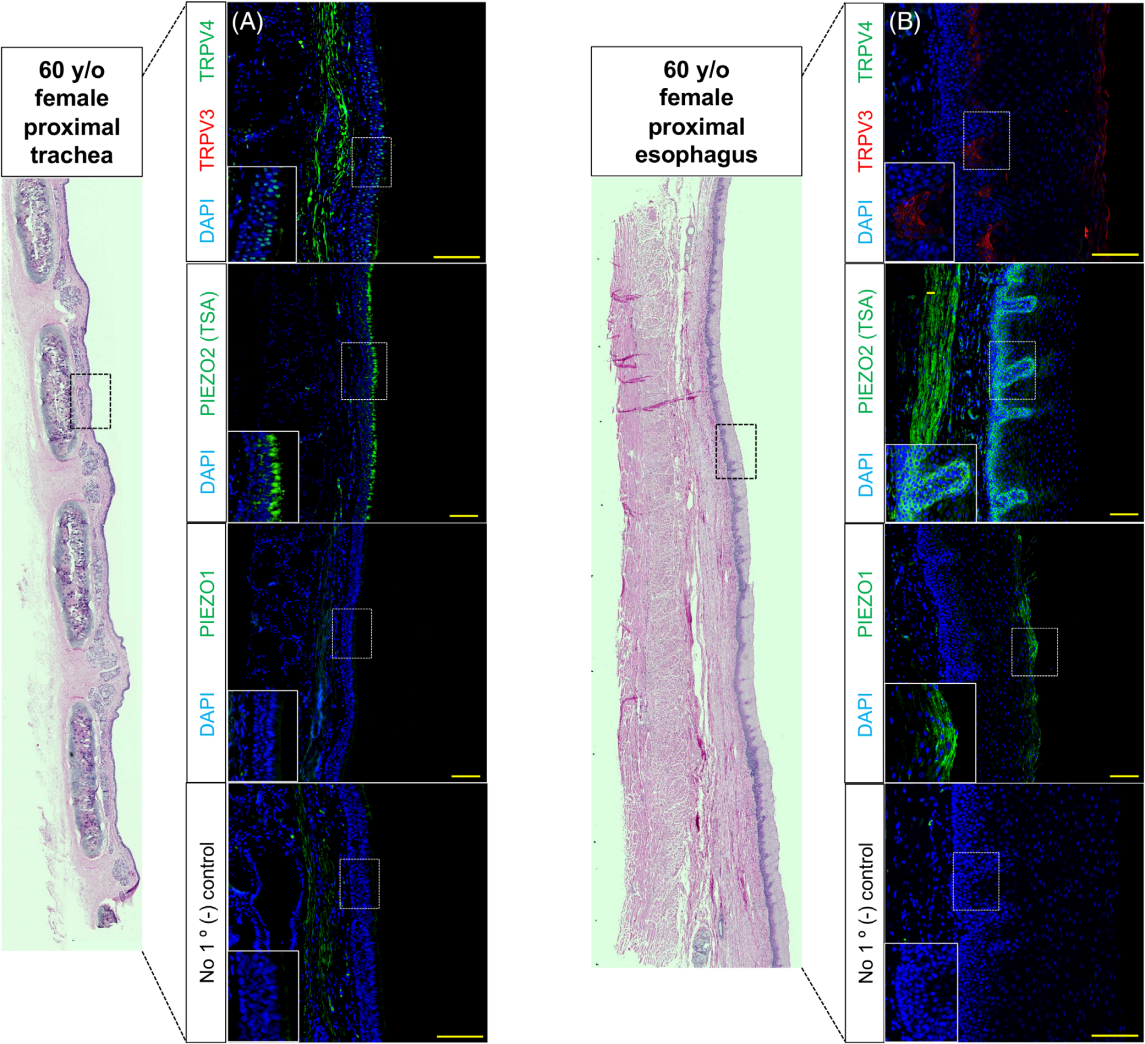
Mechano‐ and chemoreceptors in 60‐year‐old female proximal tracheal and esophageal tissue. Low magnification of hematoxylin and eosin‐stained coronal sections. (A) Immunofluorescence analysis for chemoreceptor TRPV4 (green) and mechanoreceptor PIEZO2 (green) exhibited to tracheal epithelial cells with lack of TRPV3 (red) and/or PIEZO1 (green) expression. (B) Chemoreceptor TRPV3 (red) and mechanoreceptor PIEZO2 (green) exhibited to esophageal basal epithelial stratum, in addition to, TRPV3 and PIEZO1 (green) localized to apical stratum. No positive expression was noted for TRPV4 (green) in esophagus. (A, B) Negative controls lack antiserum expressivity for all anatomic regions. DAPI is in blue. Images for TRPV3/4 taken at 30× and PIEZO1/2 at 20× magnification. Scale bar represents 100 μm

Next, we collected human vocal fold, arytenoid, tracheal and esophageal tissue samples from a 60‐year‐old female for comparison to adult 6 weeks mouse tissue. In addition to staining human laryngeal samples for TRPV3/4, PIEZO1/2 mechanoreceptors were localized. Within human vocal fold tissue, TRPV4 localized to epithelial cells of the ventricular space and subglottis (Figure 4A,B). TRPV3 was only expressed in few apical epithelia of the subglottis (Figure 4D). PIEZO2 localized to epithelia of the false vocal fold, ventricular space and subglottis (Figure 4A,B,D). There was no expression of TRPV3/4 or PIEZO2 to epithelia of the true vocal fold (Figure 4C). On the contrary, PIEZO1 localized to apical stratified squamous epithelia of the true vocal fold only (Figure 4C). Within human arytenoid mucosa, TRPV3 localized to apical stratified squamous and intermediate epithelia (transition zone), albeit, exhibited no expression to respiratory epithelium (Figure 5A–C). TRPV4 and PIEZO2 were only expressed to cells of respiratory epithelia (Figure 5C). PIEZO1 was not found in either respiratory, intermediate and/or stratified squamous epithelia of the arytenoid (Figure 5A–C). Lastly, within human tracheal tissue, TRPV4 and PIEZO2 localized to tracheal epithelium, albeit, no TRPV3 or PIEZO1 was exhibited (Figure 6A). Within human esophageal tissue, TRPV4 was not expressed, however, TRPV3 was noted to invaginations of the basal epithelium along with weak staining at the apical surface. PIEZO2 localized to epithelia lining the basal compartment, with PIEZO1 found localized to apical stratified squamous epithelia (Figure 6B).

## 4 DISCUSSION

The larynx is an organ with diverse, evolved roles in carrying out and coordinating critical functions for breathing, swallowing, cough, and phonation. This requires continuous monitoring across the lifespan via cellular and neural mucosal sensory receptors, enacting laryngeal reflex responses. Receptors have been broadly categorized as either mainly responding to chemical and/or mechanical stimuli. In this study, we initially focused on TRPV chemoreceptors, which have been largely implicated in their role in airway protection via the LCR. The LCR is present across the lifespan, beginning in the fetus and newborn to elicit glottic closure and apnea, yet in the infant and adult adapts to elicit a cough reflex. TRPV ion channels, primarily expressed on C‐fibers, have been localized to laryngeal mucosal tissues and have important chemosensory properties.^13^, ^15^ However, up‐to‐date, developmental expression patterns are unknown. We localized TRPV3/4 in murine tissue across development at E16.5, P0, and 6 weeks timepoints with direct comparison to adult human VF mucosal tissue. Protein expression of TRPV3/4 were colocalized as early as E16.5 in the mouse larynx, predominating in epithelium of the supraglottis to focal regions of the epiglottis, aryepiglottic folds and arytenoid mucosa. TRPV4 expression extended to the 6 weeks adult larynx with robust expression to posterior arytenoid mucosa, hypopharynx and anterior epiglottis. TRPV3 expression was found colocalized with TRPV4 to epithelial cells of the epiglottis, albeit, restricted to taste buds of the posterior arytenoids at 6 weeks. Previous work has demonstrated increased density of afferent laryngeal receptors to the laryngeal aspect of the epiglottis, aryepiglottic folds and arytenoid cartilages,^43^, ^44^, ^45^ with recent evidence suggesting the vital role for taste buds in laryngeal chemoreception.^24^, ^46^ Posterosuperior supraglottic distribution of laryngeal afferent receptors is thought to be evolved for relay of timely sensory information about hazards entering the airway.^5^

The posterosuperior supraglottis in humans has been shown to be highly sensate to mucosal mechanical stimulation, enacting the laryngeal adductor reflex.^4^, ^5^ Other work has also demonstrated *Piezo1* and *Trpv4* interdependence,^18^, ^19^, ^21^, ^27^ therefore, we localized mechanoreceptors PIEZO1/2 in adult human laryngeal epithelium. PIEZO1 was found only localized to apical cells of stratified squamous epithelium (true vocal fold). On the contrary, PIEZO2 localized to respiratory epithelium in regions of the supraglottis (arytenoid, false vocal fold, and ventricular space), subglottis and trachea. TRPV3/4 exhibited most robust expression to arytenoid epithelium, albeit, alike to PIEZO1/2, was selective to certain epithelia. For example, TRPV3 expressed in stratified squamous epithelium of the posterior arytenoid and esophagus, whereas TRPV4 localized to respiratory epithelia of the ventricular space, arytenoid and trachea. Most often, TRPV4 was localized to epithelia also expressing PIEZO2. Interestingly, PIEZO2 was also found localized to basal cells lining the proximal esophagus. We suspect this may have do to with the mechanical forces experienced during esophageal distension‐induced peristalsis,^47^, ^48^ however, to the best of our knowledge has not been investigated in the literature. Other work along the digestive tract, particularly in the intestines, has found that epithelial enterochromaffin cells require *Piezo2* to convert force into serotonin release.^49^, ^50^ Taken together, our data suggest species conservation of TRPV3/4 chemoreceptors, exhibiting robust expression to sensory hotspots in the human larynx such as mucosa of the arytenoid region and subglottic tracheal epithelium.

## 5 CONCLUSION

Provided its vast functionality, the larynx has evolved to provide critical and timely sensory information for airway protection. This ultimately requires mucosal surveillance by way of neural and cellular laryngeal sensory receptors, responsive to both chemical and mechanical cues. Normal physiologic reflex responses to laryngeal stimuli vary across the lifespan; critical during perinatal development for a patent airway and coordinating a safe swallow. In this study, we demonstrated murine expression of TRPV3/4 chemoreceptors at E16.5, P0, and 6 weeks developmental stages with colocalization in epithelial cells to supraglottic regions of the arytenoids, aryepiglottic folds, epiglottis, and hypopharynx. Expression patterns for TRPV3/4 between mouse and human adult stages exhibited similar regional expression patterns. Previously uncharacterized in human, we additionally localized PIEZO1 expression, selective to differentiated, stratified squamous epithelia of the true vocal fold and esophagus, while PIEZO2 expression exhibited selectivity for intermediate and respiratory epithelia of the false vocal fold, ventricles, subglottis, arytenoid, and trachea. Results suggest importance of TRPV3/4 chemosensory receptors in airway protective responses during fetal/neonatal development with continued surveillance via mechano‐ and chemoreceptors in postnatal stages. Additional insight into laryngeal sensory receptors may help to better explain LCR abnormalities observed in the pathogenesis of certain fetal/neonatal and adult sensory‐based disorders.

## FUNDING INFORMATION

This work was supported by grants NIH NIDCD R01 DC04336, and F31 DC018978.

## CONFLICTS OF INTEREST

The authors have no relevant financial or non‐financial interests to disclose.
